# Label-Free Morphology-Based Prediction of Multiple Differentiation Potentials of Human Mesenchymal Stem Cells for Early Evaluation of Intact Cells

**DOI:** 10.1371/journal.pone.0093952

**Published:** 2014-04-04

**Authors:** Hiroto Sasaki, Ichiro Takeuchi, Mai Okada, Rumi Sawada, Kei Kanie, Yasujiro Kiyota, Hiroyuki Honda, Ryuji Kato

**Affiliations:** 1 Department of Biotechnology, Graduate School of Engineering, Nagoya University, Nagoya, Aichi, Japan; 2 Department of Computer Science/Scientific and Engineering Simulation, Graduate School of Engineering, Nagoya Institute of Technology, Nagoya, Aichi, Japan; 3 Department of Basic Medicinal Sciences, Graduate School of Pharmaceutical Sciences, Nagoya University, Nagoya, Aichi, Japan; 4 Division of Medical Devices, National Institute of Health Sciences, Setagaya-ku, Tokyo, Japan; 5 Nikon Corporation, Chiyoda-ku, Tokyo, Japan; Michigan State University, United States of America

## Abstract

Precise quantification of cellular potential of stem cells, such as human bone marrow–derived mesenchymal stem cells (hBMSCs), is important for achieving stable and effective outcomes in clinical stem cell therapy. Here, we report a method for image-based prediction of the multiple differentiation potentials of hBMSCs. This method has four major advantages: (1) the cells used for potential prediction are fully intact, and therefore directly usable for clinical applications; (2) predictions of potentials are generated before differentiation cultures are initiated; (3) prediction of multiple potentials can be provided simultaneously for each sample; and (4) predictions of potentials yield quantitative values that correlate strongly with the experimental data. Our results show that the collapse of hBMSC differentiation potentials, triggered by *in vitro* expansion, can be quantitatively predicted far in advance by predicting multiple potentials, multi-lineage differentiation potentials (osteogenic, adipogenic, and chondrogenic) and population doubling potential using morphological features apparent during the first 4 days of expansion culture. In order to understand how such morphological features can be effective for advance predictions, we measured gene-expression profiles of the same early undifferentiated cells. Both senescence-related genes (p16 and p21) and cytoskeleton-related genes (*PTK2*, CD146, and CD49) already correlated to the decrease of potentials at this stage. To objectively compare the performance of morphology and gene expression for such early prediction, we tested a range of models using various combinations of features. Such comparison of predictive performances revealed that morphological features performed better overall than gene-expression profiles, balancing the predictive accuracy with the effort required for model construction. This benchmark list of various prediction models not only identifies the best morphological feature conversion method for objective potential prediction, but should also allow clinicians to choose the most practical morphology-based prediction method for their own purposes.

## Introduction

The application of recent advances in cell technologies in regenerative medicine holds great promise for revolutionizing conventional medical therapies [1]. However, the lack of assessment technology for quantitatively evaluating cell quality, in particular for revealing both the current properties and the future potentials of intact cells, is a technical obstacle to the development of quality-assured cellular products for medical use [2], [3]. Conventional methods for cellular assessment using standard techniques of molecular biology are incompatible with satisfying clinical requirements, because these methods damage cultured cells. As a result, manual microscopic monitoring, the basic and the most traditional scheme for maintaining cells, is still the most practical quality-control method for facilities that distribute regenerative cell therapies [4]–[6].

Technological advances in optical systems and image-processing technologies have changed the status of image-based data from an art, available only to experts, to a technique that can be used to generate unbiased data. Many high-content image-analysis methodologies based on imaging and image-processing technologies, especially those focused on fluorescently labeled images, have contributed to advances in drug discoveries [7]–[10]. In the field of applied cell therapy, several reports have indicated that cellular morphological information, combined with sophisticated computational modeling approaches, can serve as a descriptive indicator in evaluations of stem cells [11]–[14]. However, to fulfill the clinical requirements for producing intact cells for therapies, wider use of cell-morphology analysis methodologies that focus on label-free images should be encouraged.

In an effort to overcome these limitations of conventional methods using fluorescently labeled images, we previously performed a model case study of the label-free morphology-based prediction of the osteogenic differentiation potential of human bone marrow–derived stem cells (hBMSCs) [15], [16], using a technique that combines an automatic cell monitoring system with effective computational modeling [17]. Statistically extracted features of cellular morphologies clearly indicated that their information content can satisfactorily train computational models, not only to quantitatively evaluate current cellular status, but also to quantitatively forecast their future status, i.e., their potentials. The greatest advantage of our proposed morphology-based cell quality assessment is its non-invasiveness. As a result of this feature, our method has benefits that cannot be achieved by conventional techniques for producing cells for clinical regenerative medicine: (1) elimination of risk factors, e.g., contamination and mishandling by the operator; (2) synchronic and flexible scheduling of culture and clinical operations, for the best timing of cellular activity; and (3) repeated assessment of the same sample, by multiple criteria and at multiple times, yielding data that better reflects the complex and dynamic features of the samples. Such intelligent control of culture processes is also a key technology for process automation [18].

In this work, we expanded our previous efforts to predict single-lineage differentiation potentials [17] by pursuing five important aims: (I) Confirmation of the robustness of our method for adapting to the practical cellular variation. In our earlier work, it was not clear whether our original methodology was applicable to wider ranges of cellular variations. To investigate this issue, our data were expanded to cover eight continuous passages, ranging from very recently derived cells to those that had completely lost their doubling potential. Since a computational modeling solution for adapting to cellular variations resulting from patient diversity was already proposed in our previous work [17], our experimental design in this work was focused on cellular variations affected by culture processes, because these are the most difficult aspect of stem cells to evaluate daily. (II) Investigation of the possibility of shifting the prediction timing to the very early stage. Our previous prediction required 2 weeks of image acquisition after the differentiation process began [17]. In this study, however, we investigated whether much earlier and shorter periods were possible. In this work, only four images, obtained from the same sample repeatedly with a 24-hour interval during the first 4 days of expansion before differentiation culture, were used in the predictions. (III) Multiplication of the variations of *in silico* predictions. Compared to the previous prediction scheme [17], which could predict osteogenic differentiation potential from the same image, in this study we attempted to predict four types of potentials (osteogenic/adipocyte/chondrocyte differentiation, and population doubling time (PDT)) from the same image. Such simultaneous prediction of multiple potentials for the same cells can be achieved by processing the same image data, although the predictions are performed by four types of differently trained prediction models running in parallel. Thus, this is a trial of “overlapping” computational evaluation that can compensate for multiple immunohistochemical staining. (IV) Establishment of new conversion schemes of morphological feature usage that can achieve high predictive performance. Morphological features are the essential information generated from imaging data, and use of this information is critical in imaging-based applications. To date, however, there have been few comprehensive studies that compare the effects of different conversions of morphological features, especially in the context of label-free time-course imaging data. To reveal differences resulting from the use of various morphological features, we proposed six types of novel morphological feature conversion methods, and then compared their prediction performances in detail. To interpret the patterns of morphological features engaged in high-performance models in each differentiation lineage, we selected LASSO regression as a modeling method. (V) Quantitative comparison of morphology and gene expression in prior prediction of differentiation potential. Although morphological information has long been used as an indicator for cellular evaluation, it has remained unclear how descriptive such information really is. To quantitatively compare the performance of morphological and biological information, we directly compared the performances of predictive models using morphological features, gene expression, or both in predicting differentiation potentials from the undifferentiated state. This comparison provides a performance benchmark for our proposed morphology-based cellular potential prediction methodology, enabling complete, non-invasive, daily cellular evaluations that could support or complement evaluations that rely on conventional biomarkers.

## Results

### Construction of a dataset that relates hBMSC morphological information with differentiation potential, for the purpose of developing a model for early prediction using undifferentiated status images

**Figure 1:**
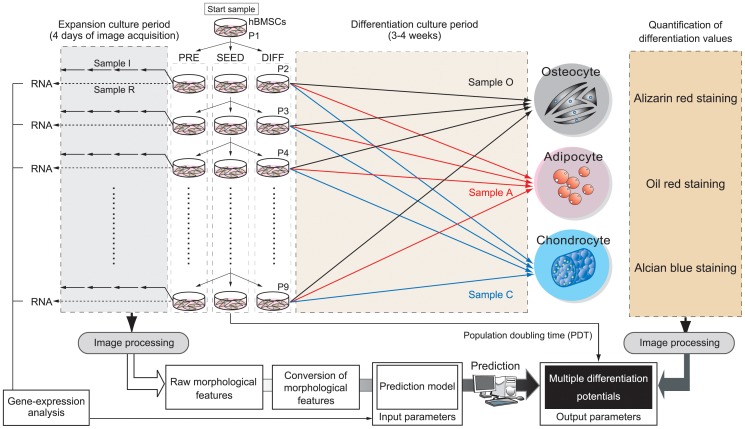
Schematic illustration of experimental setup for dataset construction for morphology-based prediction model construction. Figure S1 shows the illustration of usage of the objective morphology-based prediction model, and its major technological achievements using this dataset. The initial sample (P1) was divided into three separate culture samples (SEED, PRE, and DIFF) at each passage. SEED samples were mainly used for the continuous-passage culture until termination of growth (P9). From the cell yield at each passage of the SEED samples, population doubling time (PDT) was calculated, and taken as the experimentally determined potential. DIFF samples derived from each passage were divided into three differentiation cultures (samples O, A, and C for osteogenic, adipogenic, and chondrogenic differentiation, respectively) and grown under the indicated conditions for 3–4 weeks. The differentiation values of samples O, A, and C were experimentally quantified by individual staining protocols. The staining results were then converted by image-processing analysis to obtain the experimentally determined differentiation potentials. The three types of differentiation potentials together with the population doubling potential (population doubling time: PDT) were designated as “multiple differentiation potentials” of the hBMSCs. PRE samples consisted of sample I (for imaging) and sample R (for RNA extraction). From sample I in each passages, phase-contrast image were acquired at 24 h intervals over 4 days. Acquired images were then converted by image processing to obtain morphological features from every cell in all images (see also Fig. S2 and S3 for the details of image processing). Morphological features were statistically processed to yield transformed morphological features through data cleansing and statistical calculations, and the results were used as the input features. Sample R were subjected to total RNA extraction for gene-expression analysis. Either or both morphological features or/and gene-expression data were combined (input parameters), and arranged with the experimentally determined potentials of the hBMSCs (output parameters) to constitute training data for construction of prediction models.

To construct the morphology-based cell-quality prediction model, we first designed to prepare the dataset of hBMSCs images and their experimentally determined differentiation potential data. To assemble this dataset, three lots of hBMSCs were continuously cultured (8 passages) until their growth terminated. The range of cells was intended to mimic the wide variations in cell qualities of clinical hBMSCs. At each passage, each sample was divided into three groups: passage sample (SEED group), pre-differentiation sample (PRE group), and differentiation sample (DIFF group) (Fig. 1). Because the diversity of our cell samples was intended to mimic the clinical situation, in which a minimum cell yield is often required to meet the production criteria, the passage timing was controlled by confluency. Specifically, passage was performed when confluency exceeded 80%. Continuous passage was maintained using the SEED group. Meanwhile, the PRE group was subjected to phase-contrast microscopic image acquisition (4 days, 24-h intervals), and the DIFF samples were differentiated into three mesenchymal lineages (osteogenic, adipogenic, and chondrogenic). After long-term differentiation into the three lineages, cells were evaluated for their differentiation rate and PDT; these data were taken to represent the biological differentiation potentials. In the dataset, these potentials were linked to the morphological features measured from images in the PRE groups by machine learning using the LASSO model. Because we sought to investigate the possibility of extremely early prediction of stem cell differentiation potentials for clinical applications, we acquired our image data, which we expected to contain predictive information, before the differentiation process began. Ultimately, the full hBMSC dataset contained 24 samples of cell variants (3 lots×8 passages [P2–P9]); 80 images (5 fields of view×4 wells×4 time points) from each PRE group; and 296 experimentally determined differentiation values ( = 2×[18 images×8 passages]+[1 image×8 passages]) (Table S1).

**Figure 2:**
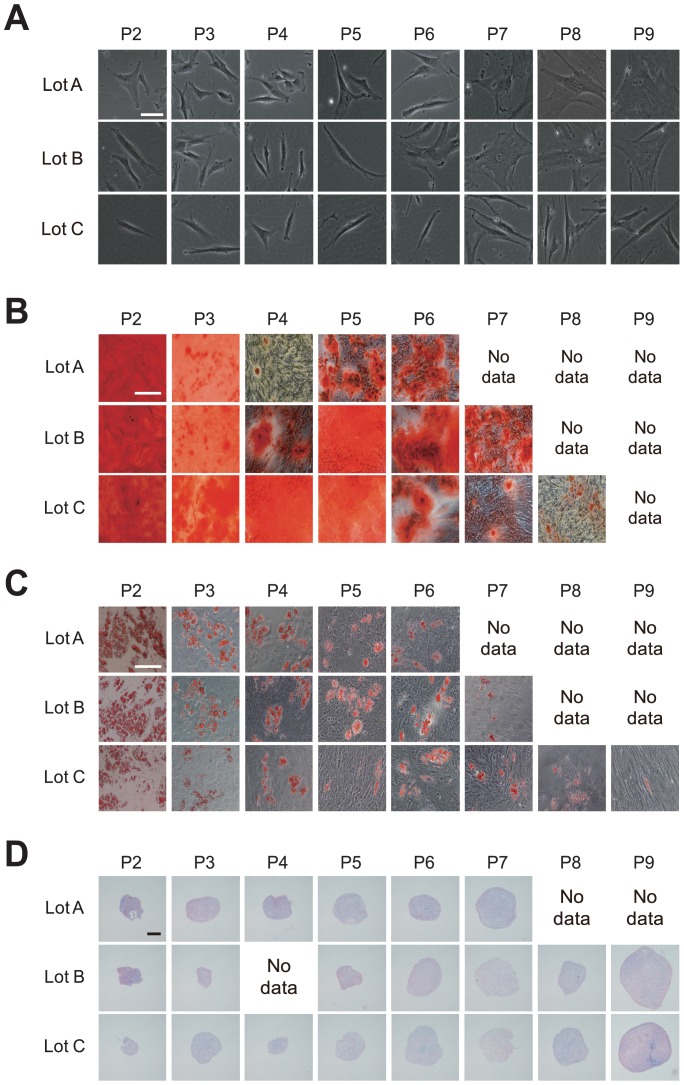
Representative morphological images of continuously passaged hBMSCs. Columns indicate passage numbers, indicated as P-number. Rows indicate hBMSC lot names. (A) Phase-contrast microscopic images (10×) prior to differentiation culture (sample I). Scale bar, 50 μm. (low-resolution cellular images shown in Figure S4) (B) Alizarin red staining after 2 weeks of osteogenic differentiation culture (sample O). Scale bar, 200 μm. (C) Oil red staining after 3 weeks of adipogenic differentiation culture (sample A). Scale bar, 200 μm. (D) Alcian blue staining after 4 weeks of chondrogenic differentiation culture (sample C). Scale bar, 200 μm. From P7–P9, near the termination of growth, differentiation samples could not be prepared for (B) and (C) because of the lack of cell numbers. In (D), when the pellet sizes were smaller than 200 μm, we declined to produce specimens from the sample on the grounds that the differentiation culture had not been successful.

By monitoring morphological changes in response to continuous passage, we observed a clear morphological transition from a spindle shape into a flat and polygonal shape, a typical indication of decay of differentiation potential (Fig. 2A). The morphological information was quantitatively extracted as morphological features, as described in the Materials and Methods section.

**Figure 3:**
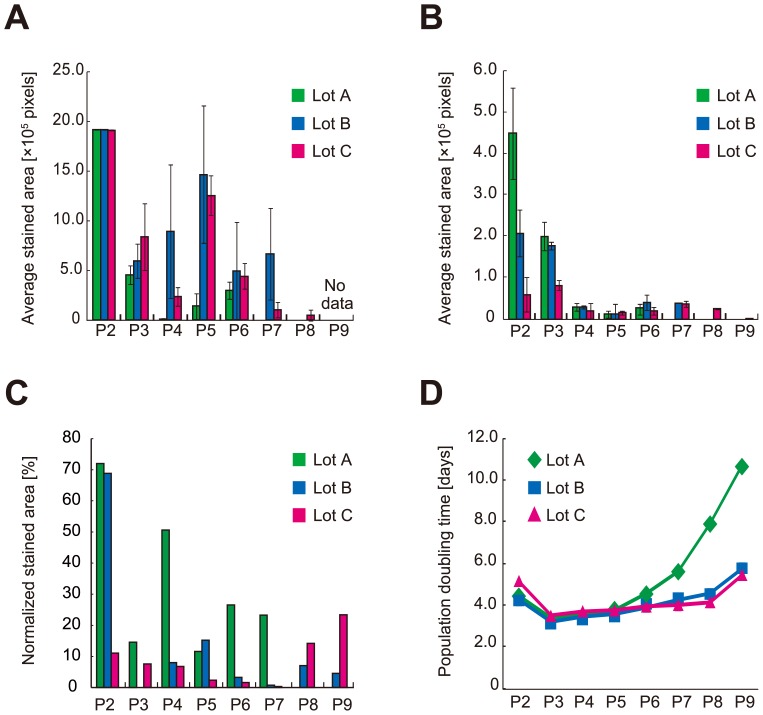
Quantified experimentally determined differentiation values and population doubling times of hBMSCs. Green bar, Lot A; blue bar, Lot B; pink bar, Lot C. Passage numbers are indicated as P2–P9. (A) Bar plots of average stained areas of Alizarin red–stained samples (*n* = 6). (B) Bar plots of average stained areas of Oil red–stained samples (*n* = 6). (C) Bar plots of stained areas in Alcian blue–stained samples (*n* = 1), normalized by their pellet size. (D) Line plots of PDT. Green diamonds, Lot A; blue squares, Lot B; pink triangles, Lot C. Error bars indicate standard deviation (s.d.).

Values related to differentiation into the three mesenchymal lineages revealed that continuous passage severely reduced the differentiation potential of hBMSCs (Fig. 2B–D, Fig. 3A–C). However, the transition patterns of differentiation potentials for the three lineages varied in a complex manner. Potentials to differentiate into adipogenic (Fig. 3B) and chondrogenic (Fig. 3C) lineages dropped rapidly, but these potentials were not correlated with the osteogenic differentiation potential (Fig. 3A). There were also variations in the changes in differentiation potentials that could be attributed primarily to differences among patients. Lot A retained its chondrogenic differentiation potential for a relatively long period, but suddenly lost it after P7 (Fig. 3C). In Lots B and C, osteogenic differentiation potential changed dramatically during continuous passage (Fig. 3A). In Lot C, adipogenic differentiation potential was sustained in any passages (Fig. 3B). Therefore, as a summary of Fig. 3B, it was realized that the tendency of differentiation potentials between patient cells can be drastically disturbed by the effect of culture process. This result indicates that simple categorization of cells by “patient information” is not effective in the clinical cell production processes, and their daily evaluation is essential.

PDT changed relatively slowly between P2 and P8 in Lots B and C (Fig. 3D). By contrast, in the case of Lot A, a rapid increase in PDT (i.e., reduction in growth rate) was observed starting at P6, indicating that this lot was sensitive to passage-related stresses (known as culture process–derived stress) triggered by both manipulations and *in vitro* culture conditions [19]. The irregular PDT increase in Lot A could be an indication of loss of differentiation potential; however, such an indication does not explain the early change in adipogenic and chondrogenic differentiation potentials in Lots B and C. These results reveal that there are no simple correlations between passage number and transition patterns. If passage numbers or PDT do not reflect changes in differentiation potential, then this information would never be sufficient to avoid a sudden quality collapse or insufficiency of cellular potential.

**Figure 4:**
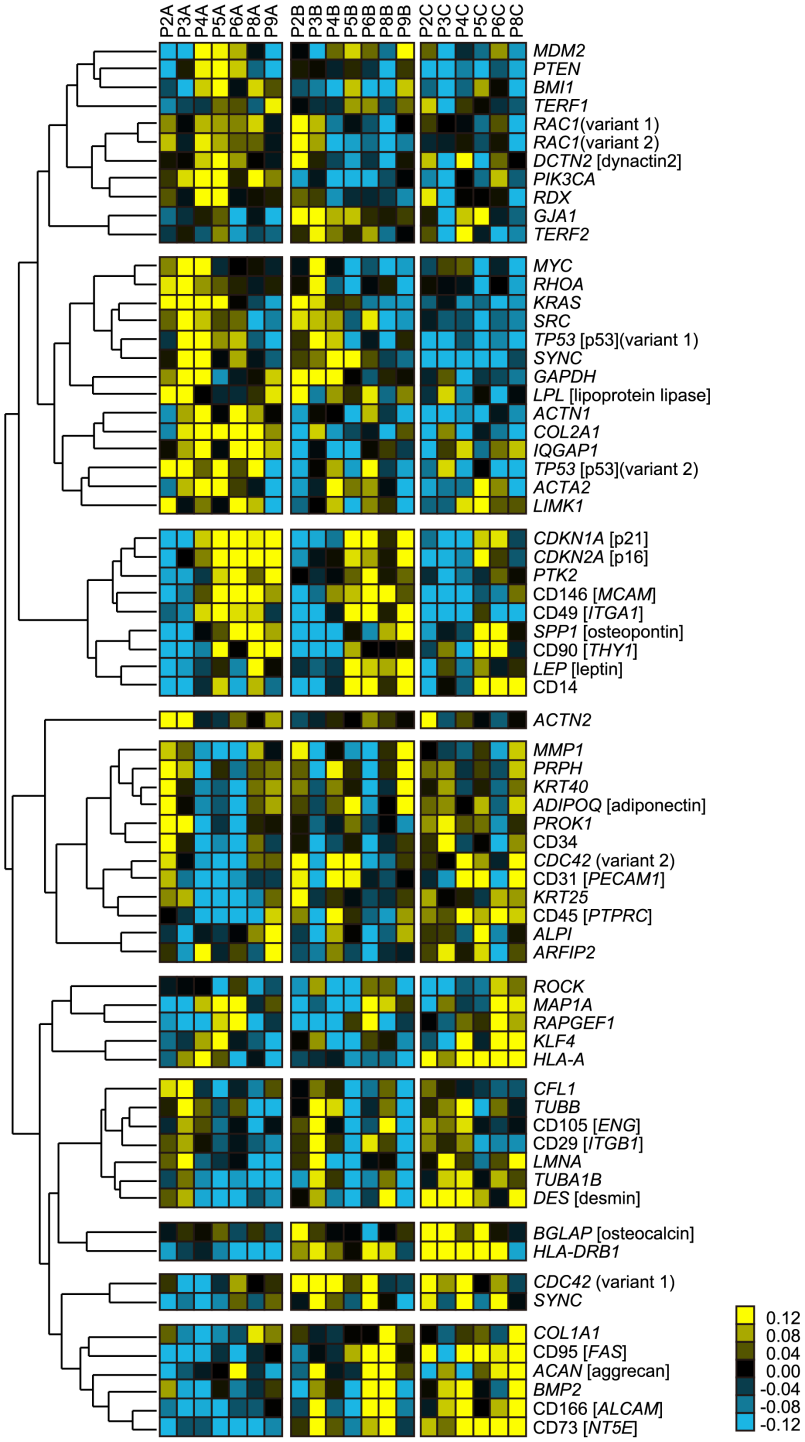
Heat map of gene-expression transitions and passage numbers. Genes were clustered by hierarchical clustering for indicating clusters that correlate to the passage number increases. The red boxed cluster is the cluster of genes that correlate to passage number within all cell lots, indicating non–patient-specific passage-related genes. The relationship between colors and normalized values of gene expression is illustrated in the explanatory heat map at lower right.

From gene-expression profiles of the earliest stage of expansion culture prior to differentiation, we found that most of the conventional differentiation markers did not exhibit clear synchronization with passage number (Fig. 4). Most of the clustered genes indicated the expression transition characteristic to certain cell lot. Therefore, there were several clusters, which partially showed synchronization to passage number in certain lot was considered to be more genes that reflect patient specific response to passages. However, in clustered genes which indicated clear synchronization with passage number (correlation coefficient>0.673) among all cell lots, either cellular senescence–related genes (*CDKN1A* [p21], *CDKN2A* [p16]) or cytoskeleton-related genes (*PTK2*, CD146 [*MCAM*], and CD49 [*ITGA1*]) were included. Since passage number significantly correlated with the decrease of differentiation potentials (Fig. 3), such passage number synchronizing gene expressions commonly observed in all cell lots were considered to be the “genetic signature of potential collapse”.

### Comparison of performances of prediction models to achieve the most balanced performance

In our previous study, we found that time courses of morphological features of cultured hBMSCs were informative in the construction of computational models aimed at forecasting future osteogenic differentiation [17]. To evaluate the multiple potentials of hBMSCs in practice, our concept of prediction had to be expanded from single-lineage to multi-lineage differentiation while retaining the ability to adapt to wider cellular variations. However, we hypothesized that in order to predict multi-lineage differentiation potential, informative morphological features and combinations thereof should be optimized for each type of differentiation. In addition, in order to increase the clinical applicability of this approach, our conceptual prediction models had to balance the accuracy with the effort (time, cost, and computational memory size) required for model preparation. Therefore, we have set our goal to define the effective construction scheme yielding the optimized prediction performance for each four different types of hBMSC potentials: potential I, osteogenic differentiation rate after 2 weeks of differentiation; potential II, adipogenic differentiation rate after 3 weeks of differentiation; potential III, chondrogenic differentiation rate after 4 weeks of differentiation; and potential IV, PDT of cells after the passages. Our objective prediction model with the newly developed techniques in this work is illustrated in Fig. S1.

**Figure 5:**
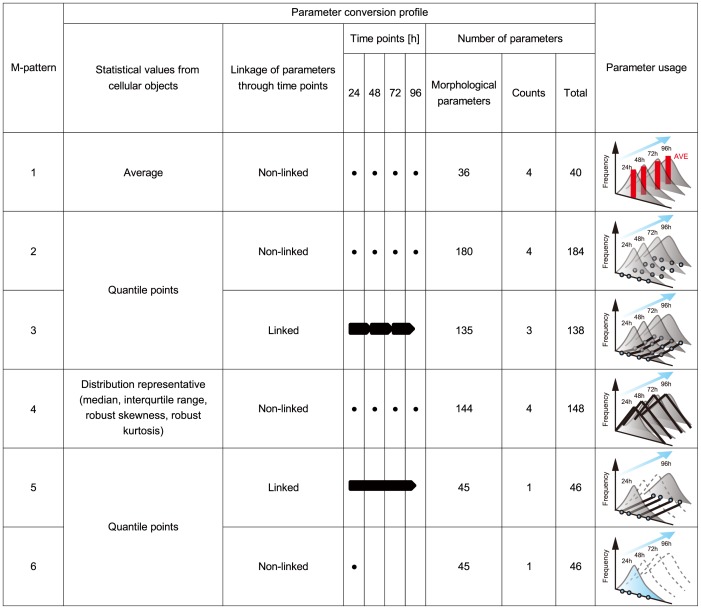
Schematic illustration matrix of prediction feature data profile and usage concepts of prediction models. Six types of morphological feature conversion methods are proposed as M-patterns. Briefly, M-patterns are numbered in order of the amount of efforts required to prepare for model construction. M-patterns 1–4 require four images at 24-hour intervals; M-pattern 5 requires two images each on days 1 and 4; and M-pattern 6 requires only one image on the first day. For parameters described as “linked”, each morphological feature is not only used as the data for each time point, but this information is also converted into the changing ratio between time points. For “non-linked” parameters, morphological features are used as they are. Averages, quintile points, and groups of distribution representatives were compared to find the best statistical parameter to represent the morphological features measured in all individual cells in an image. Therefore, M-patterns 1–4 were designed to increase the amount of information about cellular distribution for incrementing the heterogeneity of cells.

To achieve the best prediction models for these objectives, we examined nine patterns (Models 1–9) of input data usages, which critically change the users' efforts for data preparation. Model 1 was designed to be the negative control, and Models 2 and 3 were designed to compare gene expression–based predictions compared to morphology-based models. Models 4–9, consisting of five model patterns (M-patterns), were designed to compare morphological feature conversion methods by investigating the various conversion concepts and time-course data usage in morphological features (Fig. 5). The comparison of morphological features was deepened by balancing accuracy vs. feasibility of usage in the clinic. Therefore, M-patterns were numbered in the order of higher cost performance, considering the time, cost, and computational memory size involved in the model-construction process. All model performances were compared by two criteria, “scaled error rate” and “correlation coefficient”. The scaled error rate indicates the median value of prediction errors among all the samples, normalized by the actual experimental values. Therefore, low scaled error rate indicates that prediction values are relevant to the experimental values. However, usage of the scaled error rate alone is vulnerable to accidental prediction noises. Therefore, we introduced the second criterion, correlation coefficient, which evaluates the combined correlations of experimentally determined values and predicted values among all samples. These criteria are complementary: scaled error reflects differences between plots, however discards information about overall plot accuracy, whereas correlation coefficient reflects the overall similarity of measurements and predictions, however is sensitive to outliers. The combination of low scaled error rate and high correlation coefficient indicates stable performance of a given model.

**Figure 6:**
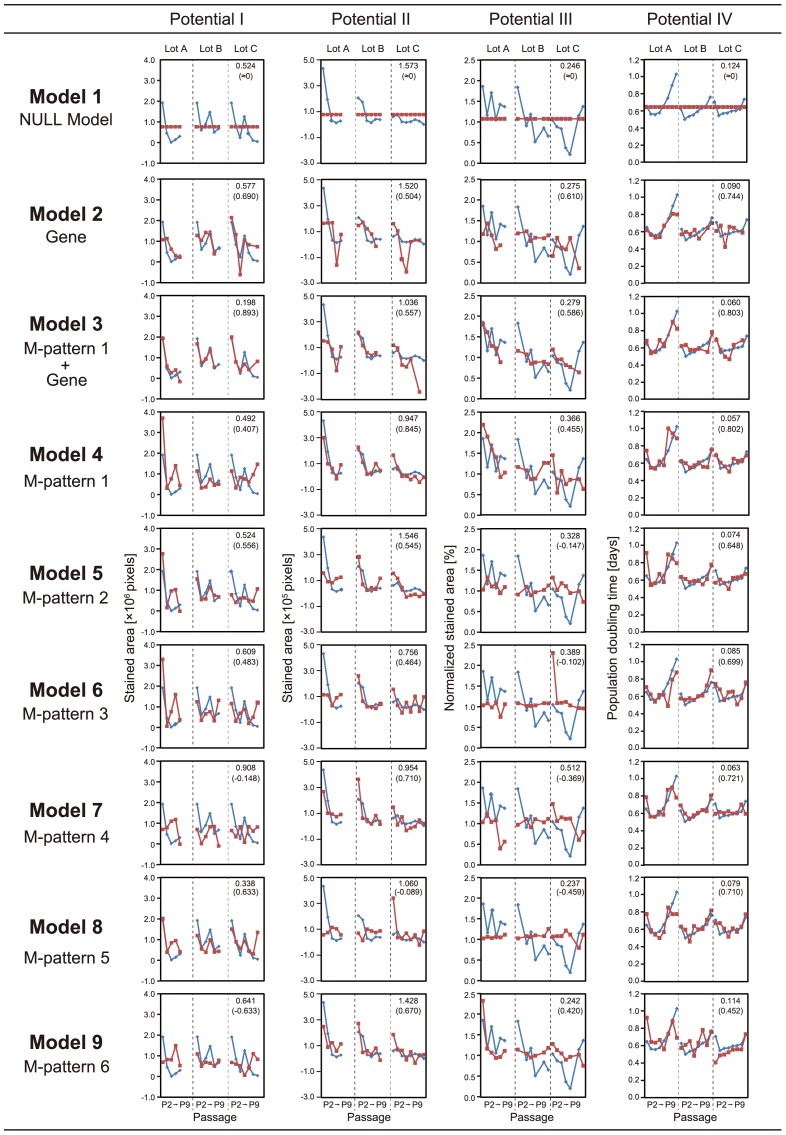
Comparisons of prediction models. For the detailed definition of “M-pattern”, see Material and Methods. Conceptual illustration of M-pattern appears in Figure 4. Potential I, osteogenic differentiation rate; potential II, adipogenic differentiation rate; potential III, chondrogenic differentiation rate; potential IV, PDT. Each matrix involving line plots consists of three columns, separated by dotted lines, representing differences among lots (Lots A, B, and C). In each column, horizontal axis represents passage numbers, from P2 on the left to P9 on the right. Upper number at the shoulder of each matrix indicates scaled error rate, i.e., the median value of prediction errors among all the samples, normalized by the experimental values. The lower number at the shoulder of each matrix indicates the correlation coefficient. Blue line plot represents the value of experimentally determined values. Red line plot represents the prediction values from the prediction models. Greater overlap between blue and red line plots and minimum differences across passages and lot differences corresponds to higher predictive performance, represented by lower scaled error rate and higher correlation coefficient.

Figure 6 depicts all prediction results. The data indicate that by using only the prior morphologies before the differentiation process, future collapses in all of differentiation potentials (potential I–IV) under continuous passage stresses can be predicted in advance. Comparisons of the transition patterns of the experimentally determined and predicted values (blue line plots and red line plots, respectively, depicted in Fig. 6) revealed that all cellular properties were predicted with reasonable accuracy. Furthermore, in contrast to our previous study that used all morphological data from 14 days of differentiation culture period [17], the predictive performance was enhanced in this study using morphological data collected only from the first 4 days before the differentiation.

For prediction of potential I (osteogenic differentiation rate), the best prediction accuracy was achieved by Model 3, which utilizes both morphological features (M-pattern 1) and gene-expression profiles. Compared to the scaled error rate of the NULL model (Model 1), the performance of Model 3 can be expressed as 2.6-fold more accurate. In the sense of cost-efficiency of model construction, Model 8, which utilizes only the morphological features from 24 h, had a reasonably high predictive performance (scaled error rate = 0.338).

For predicting potential II (adipogenic differentiation rate), morphology-based models such as Models 4, 6, 7, and 9 yielded extremely high predictive performance. Model 4 achieved the best accuracy, and Model 7 was the best model at the lowest cost (scaled error rate = 0.954).

For predicting potential III (chondrogenic differentiation rate), most of the models could not significantly outperform the NULL model. However, Model 9 had fairly accurate predictive performance.

For predicting potential IV (PDT of cells after repeated passages), most of the models had very high predictive accuracies (scaled error rate<0.09). The best performance was achieved by Model 4, which utilizes only morphological features.

Although morphology-based prediction models (Models 4–9; M-Patterns 1–5) had consistently high overall performances in predicting various potentials, the use of direct biological information (i.e., gene-expression information including conventional differentiation markers) did not dramatically improve the predictive performance (Model 2 in Fig. 6). From the interpretation of parameter usages in LASSO models (Table S4–S7), we found that cytoskeleton-related genes were more frequently involved than differentiation markers in the prediction models (Models 2 and 3). This result is a biological confirmation that morphological genes are more informative than our selected subset of differentiation marker genes for prediction of differentiation potential, and explains the high performances of models that use only morphological data (Models 4–9).

## Discussion

To replace human estimations of cell quality in the production of cells for cell-based therapies, we examined the performances of machine-learning models in predicting the quantitative rates of multi-lineage differentiation after long-term differentiation, using data from undifferentiated label-free images of hBMSCs. The novel advancing technological points achieved in this work are illustrated in Fig. S1. From images collected during the first 4 days of expansion culture before differentiation, the morphological features of each cell in the images were individually measured and converted into various morphological metrics that represented the statistical morphological profiles of the group of cells. These features were then used to train computational models that forecast the experimental results collected 2–4 weeks after the differentiation. Advancing from our previous success in predicting the single-lineage differentiation potentials of hBMSCs [17], here we showed that the best predictive results for all differentiation potentials (i.e., the differentiation rates into three lineages and their PDTs) can be obtained at the same time, even in the early stage before differentiation, using selected morphological features. In these comparisons, we addressed three technical questions, with the aim of identifying the most practical scheme for obtaining such cell-quality prediction models in clinical facilities. First, can morphology-based prediction methods be expanded to the prediction of multiple differentiation potentials? Second, is morphological information (i.e., indirect phenotypic signals) of greater use than gene-expression information (i.e., direct biological signals) in predicting the qualities of hBMSCs? Third, how far can we optimize model performance by selecting the appropriate conversion and combination of information from the time-course morphological features?

To our great surprise, considering the current lack of comparable evaluation methods, most of the examined prediction models using only morphological features showed practically useful performance in multiple predictions (Fig. 5 and 6). Even with the Model 9 (M-pattern 6, using morphological features obtained only from the first day of expansion culture), the multi-lineage potential prediction was available. Practically, potential II (adipogenic differentiation rate after 3 weeks) can be predicted with high accuracy using only morphological data from the first 4 days of culture. Both potentials I and III (osteogenic and chondrogenic differentiation rates) could also be predicted with reasonable accuracy from the early morphological data. In addition to differentiation rates, future PDT following repeated passages can also be predicted with high accuracy using only morphological features. These results strongly indicate that it will be possible to develop practical methods for cell assessment that are multiple, rapid, cheap, non-invasive, and significantly more effective than conventional staining-based assessment techniques. Our models' performance indicate that such novel predictive methods will enjoy several advantages: (1) non-invasiveness, i.e., avoiding damage to patients' cells; (2) synchronism, repeated quality evaluation throughout the culture period for all patients; and (3) multivalent consideration of the same sample, i.e., multiple quality assessments can be performed with the same sample, which is not possible when using data obtained by destructive methods such as fluorescently labeled imaging analysis.

The quantitative predictions made possible by these methods will permit prior evaluation of cellular fate, which will in turn facilitate scheduling of cell-therapy operations in the clinic. As shown in Fig. 3, most of the transition events in hBMSC potentials were abrupt, and would be nearly impossible to estimate the future linearly from the present result plots. Therefore, conventional cell-assessment techniques could never outperform quantitative prediction methods for hBMSC quality assessment. Our results thus provide a successful example of the use of machine-learning models to model biological information and generate output that can overcome a major practical problem in clinical cell therapy.

Taken together with the non-linear correlation of conventional marker gene-expression levels with passage numbers (Fig. 4) and the predictive performance of models (Models 2 and 3 in Fig. 6), we concluded that morphological data from the early stage of culture are more useful than measurements of conventional markers in forecasting future quality disruptions. In some cases, gene-expression measurement enhanced morphological predictions, when an early gene marker such as *SPP1* [osteopontin] occasionally function as extreme early osteogenesis predictor (Model 2, Potential I prediction in Fig. 6). However, differentiation gene markers are not always promising to function as extreme early predictor in the undifferentiation stage. By introducing LASSO modeling into this work, the combinational effects of parameters can be interpreted in our models (Fig. 6). In particular, by interpreting the parameter usages chosen through automatic exploration of the best LASSO model formula, it was possible to clearly detect the correlation of cytoskeleton-related and senescence-related genes with the decrease of potentials (Table S4–S7). Such correlation was supported by previous studies showing that the TGF-β signaling cascade links cellular quality collapse with morphological changes [20]–[22].

Compared to the successful predictions of adipogenic differentiation potential and PDT, the predictive performance for osteogenic and chondrogenic differentiation could not be increased by altering the modeling techniques. We believe that the main reason for this limitation on performance did not reflect a shortcoming of our method. Machine-learning performance relies heavily on the quality of training data. In this work, we are uncertain of the quality of our ‘teacher signal’ data, i.e., the converted data from conventional staining assays for evaluations of differentiation potential. In practice, the staining technique is usually used as only one aspect of differentiation confirmation, but is not commonly used for strict quantitative analysis. A growing body of evidence describes the quantitative use of immunohistochemical staining results in high-content analysis, analogous to the way in which we converted the staining results into numerical values. However, our machine-learning model results show that only Oil red [23], but not other staining values, results in excellent performances. In addition we have identified a few critical sources of experimental noise that might partially explain the comparatively poorer performance of osteogenic and chondrogenic differentiation. For example, in osteogenic staining with Alizarin red [24], small parts of stained cells tend to be ripped from the plate during the washing process, resulting in larger deviations within some wells. Similarly, in chondrogenic staining, the pellet size and its slice position greatly affect the staining level, resulting in larger deviations within some samples. Hence, the large difference between prediction models suggests that reproducibility and signal-to-noise ratio of staining results must be carefully examined in order for modeling to be effective. In other words, if one can introduce more stable staining, the prediction models should perform better.

Our expression data regarding other types of genes lead us to expect that effective gene combinations could be defined as new quality assessment markers (Fig. 4B, 4C). The interpretation of weights of LASSO regression models can provide insights regarding essential parameters that contributed to successful predictions (Table S4–S7). For prediction of potential I (osteogenic differentiation rate), a combination of morphological features from the whole pre-differentiation period (days 1–4), together with expression of the cytoskeleton-related genes (*RAC1* [25] and *RHOA* [26]) and the early osteogenic marker *SPP1* [osteopontin] [27], were weighted. The decision to weight these genes reflected previous reports of interactions between osteogenic marker genes and cytoskeleton genes [27]. Together, the selected morphological features support the commonly observed flat and expanded cellular morphology of hBMSCs, known as an indication of bone differentiation. For prediction of potential II (adipogenic differentiation rate), relative hole area (the morphological feature that describes the “roughness” of the cell surface) and inner radius (a reflection of polygonal and tentacle-like features in the cellular periphery) from the whole pre-differentiation period (days 1–4) were weighted. This result can be interpreted to mean that the continuous evolution of hBMSCs during the expansion period toward a jagged morphology is the signature of adipogenic potential. For prediction of potential III (chondrogenic differentiation rate), a shape factor (specifically, the roundness of cells) on day 1, but not throughout the whole period of expansion culture, was weighted. This can be interpreted to mean that very early roundness of hBMSCs can indicate the potential for chondrogenesis. For prediction of differentiation potential before induction, morphogenic markers are sufficient because these data contain both time-course and multi-parametric information. However, in microarray experiments, we identified four genes related to osteogenic differentiation (*ALPI*, *BMP2*, *BGLAP* [osteocalcin], *SSP1* [osteopontin]), three genes related to adipogenic differentiation (*ADIPOQ* [adiponectin], *LEP* [leptin], and *LPL* [lipoprotein lipase]), and one gene related to chondrogenic differentiation (*ACAN* [aggrecan]). From this background, these gene profiles were not sufficient to explain the difference in differentiation potential. As it happens, in prediction of potential I, *PIK3CA* and *SPP1* [osteopontin] were useful markers even before differentiation induction; however, for the other two cell types, there were no critical marker genes. For prediction of potential IV (PDT of cells after repeated passages), both gene-expression profiles and morphological features were weighted. Cell cycle–related genes such as *TP53* [p53] [28] and *CDKN1A* [p21] [29], [30], actin-related genes such as *ACTA2* [31] and *IQGAP1* [32], and the cellular senescence–related gene *CDKN2* [p16] [33]–[35] were heavily weighted in the best prediction models. Such gene selection reflects reports that indicate a correlation between senescence and cytoskeleton gene cascades in hBMSCs [19], [36]. The weighted morphological features (total area, inner radius, and fiber length) reflect the common culture sense of regular size and slenderness during the expansion process are markers of active hBMSCs [19], [37].

To confirm the utility of our proposed method in clinics, its adaptive performance in the context of the cellular diversity is an important criterion. Cellular diversity derived from patient diversity should be the first concern; we have previously investigated a new modeling scenario, designated as the “ongoing patient scenario”, as one strategy for adapting to such diversity [17]. In this scenario, we proved that inclusion of new patients' own morphological features in prediction models can greatly enhance the prediction accuracy for new patients. Thus, this proposed method avoids attempting to adapt to all cellular variations arising due to patient diversity, which is ideal but impossible, and instead seeks to ‘re-train’ the prediction model upon the arrival of each new patient, thereby allowing the system to adapt to new morphologies. Such re-training is feasible with our proposed regression models, and it is also feasible at various times in the clinic, because there are various opportunities to obtain images of primary cells before making critical potential predictions. Therefore, in this study, we attempted to optimize the adaptive performance of our model in the context of cellular diversity arising due to culture processes. In contrast to patient diversity, such culture process–derived diversity may expand during the cell production process, and may therefore require daily monitoring with non-invasive methods. Our results showed that even an extraordinarily diverse group of samples could be modeled feasibly using only morphological features. In addition, because our model performance is a summary of detailed cross-validation results combined with our ongoing patient scenario, we believe that our results provide a reliable performance benchmark reflecting robustness.

Throughout this work, we have successfully demonstrated models for predicting multiple future qualities of hBMSCs. Such models address an urgent need on the part of facilities that provide cell-based therapies. With similar objectives, Moghe's group and Gantenbein-Ritter's group have reported encouraging studies that reveal the effectiveness of multidimensional morphological parameter modeling in the evaluation of stem cell differentiation potentials [11]–[13]. However, in spite of the effectiveness of these strategies, such quantitative and bioinformatic technologies are still not fully utilized in clinical cell therapies. To advance image-based cellular evaluation technologies as a reliable supportive option in regenerative medicine, it will be necessary to perform more studies that connect computational technology with stem cell biology. We believe our morphology-based modeling approach will contribute to new technological developments in regenerative medicine. It has been known that cell shape takes a part in the regulation of biological processes, such as proliferation and differentiation [27], [38]–[40]. Although our main focus was to utilize such morphological information as “a signature of biological reflection” instead of investigating its meaning, our gene expression analysis and LASSO model interpretation have revealed the involvement of previously known cell shape-regulatory proteins, such as RHO-related proteins [27]. Therefore, we believe that our morphology-based modeling approach will contribute not only to the new technological developments in regenerative medicine, but also for deeper understanding of morphology effect in stem cell biology.

## Materials and Methods

### Cells and cell culture

Three lots of hBMSCs were purchased from Lonza (Walkersville, MD, USA): Lot A (lot number 8F3211, Black, Male, 18 year-old), Lot B (lot number 8F3434, Caucasian, Male, 22-year-old), and Lot C (lot number 8F3560, Hispanic, Female, 24-year-old). All cultures were maintained in MSCGM (Lonza) supplemented with BulletKit (Lonza), and sub-culture was performed with 0.025% trypsin solution (Life Technologies, Inc., Carlsbad, CA, USA). To expand the cell populations, each lot was continuously sub-cultured at 7–21-day intervals until the termination of cell growth. Passage timing was controlled by confluency. Specifically, passage was performed when confluency exceeded 80%, and we obeyed the supplier-recommended cell seeding density for Poietics™ human mesenchymal stem cells (Lonza). From all cell variants, P2–P9 samples were used. At each passage, cells were divided into three groups: passage sample (SEED group), pre-differentiation sample (PRE group), and differentiation sample (DIFF group). Sample I was subjected to an image-acquisition step. Sample R was subjected to RNA extraction for gene-expression profiling. The PRE groups represent the intact differentiation potentials of each hBMSC sample before the subsequent long-term differentiation process. The DIFF group was subdivided into three differentiation samples: Sample O for osteogenic differentiation, sample A for adipogenic differentiation, and sample C for chondrogenic differentiation. All differentiation cultures followed the protocol for Poietics Human Mesenchymal Stem Cells (Lonza). Briefly, samples were differentiated for 2 weeks (sample O), 3 weeks (sample A), or 4 weeks (sample C). See also Fig. 1 for the experimental scheme.

### Image acquisition

For sample I, 10× magnification images at the exactly same positions (five fields at the center and four neighboring images separated by vertical displacements of 2.2 mm) were obtained every 24 hours for 4 days by phase-contrast microscopy on an X71 instrument regulated by an electric x-y stage (Olympus, Tokyo, Japan) (Fig. 1). The image data were gray-scale, 8-bit, 1360×1024 TIFF format. Focusing was semi-automatically defined by the original regulation journal run by MetaMorph (Molecular Devices, Sunnyvale, CA, USA). In total, 1,920 images ( = 5 view fields×4 replicates of wells×3 lots×4 time points×8 passages) were stored in the image database.

### Image processing

All phase-contrast microscopic images were processed using MetaMorph (Molecular device) with an original combination of image-processing filter sets (Figure S2 and Table S3) to measure morphological features of cells. In the binarization process, a single universal threshold was applied to all images. The universal threshold was defined as the threshold that provided the minimum error between manually determined cell number and the recognized total object counts (obtained using that threshold) among 30 images picked randomly from all lots and time points. After binarization, all individual objects in each image, consisting of cells and noise (non-cell objects), were measured using the integrated morphometry analysis function to measure nine morphological features: (1) breadth, (2) elliptical form factor, (3) fiber breadth, (4) fiber length, (5) hole area, (6) inner radius, (7) relative hole area, (8) shape factor, and (9) total area (detailed in Figure S3). In addition to morphological features, cellular object counts were added as the tenth feature. The morphological features were carefully selected in the MetaMorph measurement settings by clustering analysis, in order to eliminate problems arising from multi-colinearity. From this data, which consisted of object IDs and the corresponding morphological features, the noise (non-cell objects) was then removed by a noise-reduction algorithm developed in-house before subsequent analysis. The object morphological data obtained from each PRE sample summarized nearly 20 images consisting of 2,000–5,000 cellular objects. The morphological feature information representing these groups of objects was characterized by various statistical values, including average (AVE), quantile points (10, 25, 50, 75, and 90%), median (MED), interqurtile range (INT), robust skewness (SKEW), and robust kurtosis (KURT) (see detail in Fig. 5). Such statistics related to morphological values were obtained from four time points (24, 48, 72, and 96 hours). Six patterns of morphological feature sets (M-patterns 1–6) were designed to examine their effects on predictive performance (Fig. 5). M-pattern 1 (non-continuous average information), 40 features: AVE of nine morphology features at all four time points. M-pattern 2 (non-continuous quantile information), 184 features: five quantile points of nine morphology features at all four time points. M-pattern 3 (short continuous quantile information), 138 features: ratio of five quantile points of nine morphological features within three intervals between four time points. M-pattern 4 (non-continuous distribution pattern), 148 features: MED, INT, SKEW and KURT of nine morphological features at all four time points. M-pattern 5 (long continuous quantile information), 46 features: ratio of five quantile points of nine morphological features within one interval between 0 h and 96 h. M-pattern 6 (short non-continuous quantile information), 46 features: five quantile points of nine morphological features at 24 h only.

### Quantitation of osteogenic differentiation rate

Calcium accumulation of differentiated samples was determined by Alizarin red staining [24] with some modifications. Cells were fixed with 70% ethanol for 1 hour, washed, and stained for 10 min with 40 mM alizarin red S solution (pH: 4.2). The quantitation of staining results was summarized by image-based measurements of the red pixels from 18 images (3 view fields×6 replicate wells, 1600×1200, color, RGB, jpg file) collected using a DP21 CCD camera (Olympus). The color image measurements were processed using MetaMorph (Molecular Devices) to extract the red pixels. The count of red pixels was taken as the quantitated value of the osteogenic differentiation rate.

### Quantitation of adipogenic differentiation rate

Samples subjected to adipogenic differentiation were stained with Oil red S [23]. Briefly, cells were fixed with 4% paraformaldehyde (PFA) for 1 hour, washed, and stained for 30 min with Oil red S solution. The quantitation of staining results was summarized by image-based measurements of the area of stained droplets from 18 images (3 view fields×6 replicate wells, 1600×1200, color, RGB, jpg file) using a DP21 CCD camera. The stained lipid droplets were identified using MetaMorph (Molecular Devices), and their total pixel area was taken as the quantitated value of the adipogenic differentiation rate.

### Quantitation of chondrogenic differentiation rate

Pellet samples differentiated into chondrocytes cultured under the chondrogenic differentiation condition were stained with Alcian blue [41]. Briefly, cells were fixed with 4% PFA for 1 hour, embedded in paraffin, and sliced into 20-μm sections. All sliced tissue samples were stained on the same day according to a conventional Alcian blue protocol. The quantitation of chondrogenic differentiation rate was obtained from image-based measurements of 1 image (1600×1200, color, jpg file) collected using a DP21 CCD camera. The area of Alcian blue staining was identified using MetaMorph (Molecular Device), and the total number of pixels was measured. To control for pellet size, the number of pixels staining positive for Alcian blue were normalized against the total pellet size (in pixels), and the resultant value was taken as the quantitated value of the chondrogenic differentiation rate.

### Quantitation of population doubling time (PDT)

At each passage, the total cell number was counted to obtain the PDT [42].

### Gene expression measurement

Total RNA was extracted using the RNAprotect Cell Reagent (Qiagen, Hilden, Germany) from sample R at confluence. RNA samples from P2–P9 were applied to the custom designed gene chip GenoPerl (Mitsubishi Rayon, Kyoto, Japan) (gene list in Table S2). The gene chip assay and analysis were carried out according to the manufacturer's protocols, and the data was used for prediction modeling. For global gene-expression analysis, expression levels of all genes were scaled by standard normalization between arrays and genes. The clustering heat map was created by Cluster 3.0 (http://bonsai.hgc.jp/~mdehoon/software/cluster/software.htm#ctv) and Java Tree View (http://jtreeview.sourceforge.net) with some modifications.

### Prediction model construction

The LASSO regression model was selected for modeling the relationships between morphological features and experimentally determined differentiation potentials. The detailed modeling process was previously described [17]. Briefly, the LASSO regression model is a penalized regression model that is widely used in the statistics and machine-learning literatures [43]. By using LASSO, one can find the linear combination of input features that best predict the teaching signal. LASSO tends to induce a sparse linear model, i.e., it can also select a set of input features that are useful for predictive purposes. The entire LASSO model-building process is automatic, including the model-selection process using leave-one-out cross-validation, and the relationships between morphological features and experimentally determined values can be represented as a simple and robust linear model. For model training, a total of 24 samples (P2–P9 in all three lots) were used as the dataset. For input features, six patterns of morphological features (M-patterns 1–6) were assigned as non-invasively obtained information for cell-quality prediction. For teaching signal information, four types of experimentally determined differentiation potentials were used to train models: Potential I, osteogenic differentiation potential (Osteo), osteogenic differentiation rate determined by Alizarin red; potential II, adipogenic differentiation potential (Adipo), adipogenic differentiation rate determined by Oil red staining; potential III, chondrogenic differentiation potential (Chondro), chondrogenic differentiation rate determined by Alcian blue staining; and potential IV, population doubling time (PDT)[42] from the growth rate over continuous passages. For the relative comparison of prediction performance, a NULL prediction model (NULL model) was constructed. The NULL model is defined as the prediction model that sets the average value of all experimentally determined values as the threshold. This imitates the case of a poor prediction, and is easily established in practical experiments. Simply put, the NULL model is a negative-control model, similar to a random-guessing model restricted to one threshold value. Furthermore, gene-expression data were also assigned in the prediction model, as rivals of morphology-based cell-quality prediction models. From the custom microarray measurements, 69 gene-expression profiles were used as additional input features in the prediction modeling. Two types of prediction models were constructed: expression data for 69 genes in combination with the M-pattern 1 feature set, and the other was the expression data for 69 genes without morphological features. In total, 36 prediction models (9 types of input feature sets×4 types of differentiation potential predictions [3 lineages and population doubling time]) were constructed (Fig. 5).

## Supporting Information

Figure S1 **Conceptual illustration of usage and technological achievements of label-free morphology-based prediction of multiple differentiation potentials.** A user can obtain three advantageous profits from our investigated method; (1) An early prediction, even from the images from the undifferentiation period to predict the final result after differentiation. Such prediction timing is designed to be fastened in this work, by the examination of the effect of early and sparse cellular images for future prediction. (2) An effective morphological feature conversion method, which can maximize the objective prediction of certain potential. Such morphological feature conversion method is comprehensively examined in this work, to reflect the meaning of heterogeneous nature of cells and their time-course changes by various ideas of morphological feature calculations. (3) A multiple simultaneous prediction for same image. In our method, four types of potential prediction model are constructed, and provide results at the same time for one image. Such paralleled prediction concept enables “overlapping” multiple evaluations of cells with non-invasive manner.
(TIF)

Figure S2 **Schematic procedure of image processing and data processing.** (A) The procedure listed as Filter 1–4 in Table S3 is illustrated. (B) The procedure listed as Filer 5–6 in Table S3 is illustrated. Especially, the illustration describes the detail of cell measurement and their data processing scheme followed by Filter 6 processing. As shown in the figure, all cells in the images are measured as data consist of “group of cells”, and their distribution is used to calculate statistic values to describe such “group of cells”. Through the process, ells are measured individually by morphological indices; however our final morphological features reflect the information of “group of cells”. In other words, our morphological features contain the information of “heterogeneity” of “group of cells”, which strengthen our prediction model.
(TIF)

Figure S3 **Detailed definition of the basic nine morphological features used for cell measurements.** Formulas and schematic illustration of morphological features are presented in detail. The nine features are carefully selected to represent independent information, with the aspect of low-correlating parameters, for stabilizing prediction models which utilize morphological features from label-free phase contrast images.
(TIF)

Figure S4 **Low-magnification images of cells in** **Figure 2A** **.** The low-magnification images provides overall image of cellular morphological profile and its distribution.
(TIF)

Table S1 **Dataset profile used for prediction model construction.**
(XLSX)

Table S2 **Gene list in custom designed gene chip microarray.**
(XLSX)

Table S3 **Image processing scheme.**
(XLSX)

Table S4 **Parameters and weights explored to contribute in prediction models for Potential I.**
(XLSX)

Table S5 **Parameters and weights explored to contribute in prediction models for Potential II.**
(XLSX)

Table S6 **Parameters and weights explored to contribute in prediction models for Potential III.**
(XLSX)

Table S7 **Parameters and weights explored to contribute in prediction models for Potential IV.**
(XLSX)
