# Supplementary figures and images for: Label-Free Morphology-Based Prediction of Multiple Differentiation Potentials of Human Mesenchymal Stem Cells for Early Evaluation of Intact Cells
Source: PLoS One. 2014 Apr 4;9(4):e93952. doi: 10.1371/journal.pone.0093952 (PMC3976343; doi:10.1371/journal.pone.0093952)

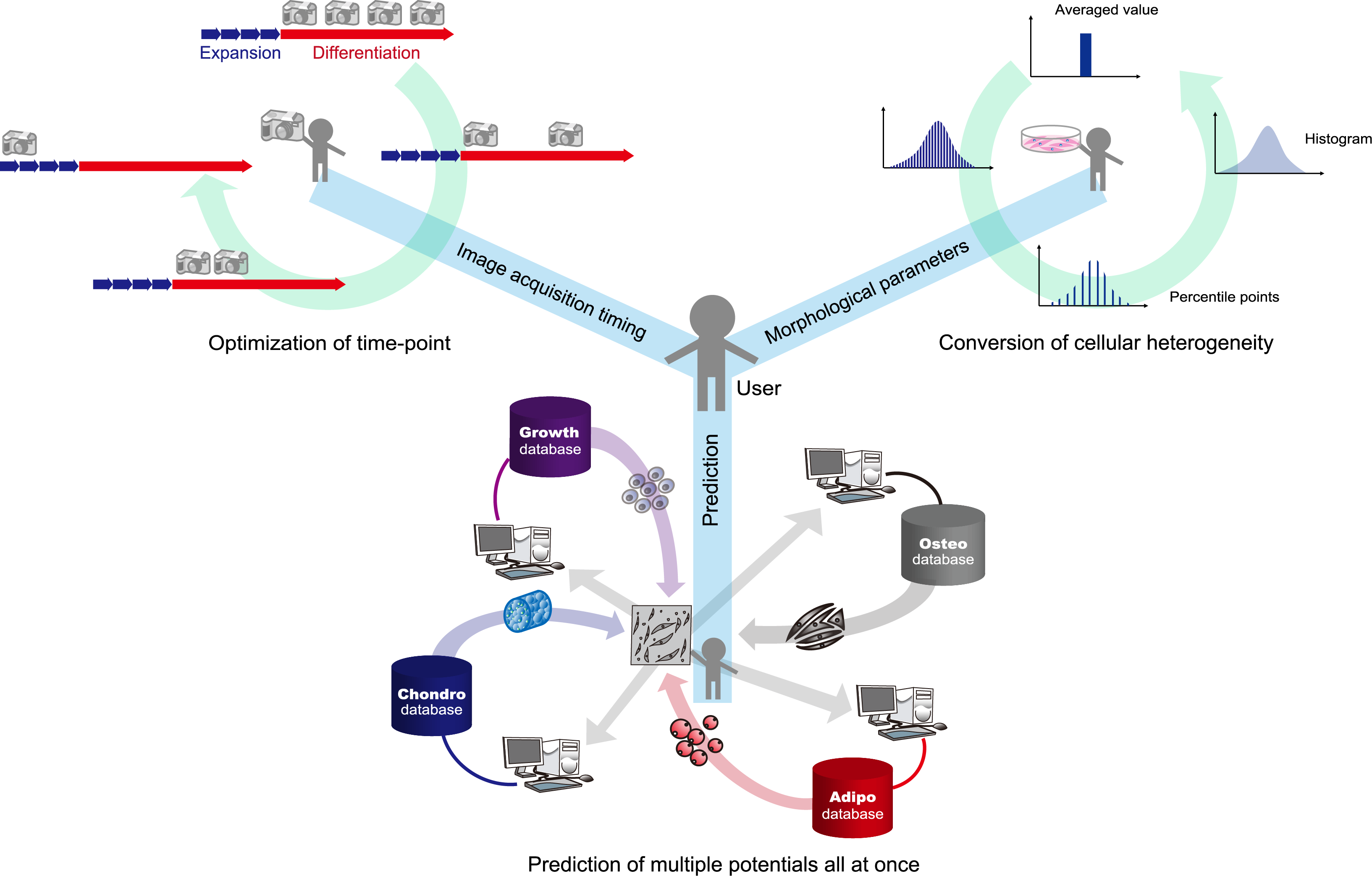

Supplement: Figure S1 — Conceptual illustration of usage and technological achievements of label-free morphology-based prediction of multiple differentiation potentials. A user can obtain three advantageous profits from our investigated method; (1) An early prediction, even from the images from the undifferentiation period to predict the final result after differentiation. Such prediction timing is designed to be fastened in this work, by the examination of the effect of early and sparse cellular images for future prediction. (2) An effective morphological feature conversion method, which can maximize the objective prediction of certain potential. Such morphological feature conversion method is comprehensively examined in this work, to reflect the meaning of heterogeneous nature of cells and their time-course changes by various ideas of morphological feature calculations. (3) A multiple simultaneous prediction for same image. In our method, four types of potential prediction model are constructed, and provide results at the same time for one image. Such paralleled prediction concept enables “overlapping” multiple evaluations of cells with non-invasive manner. (TIF) [file pone.0093952.s001.tif]

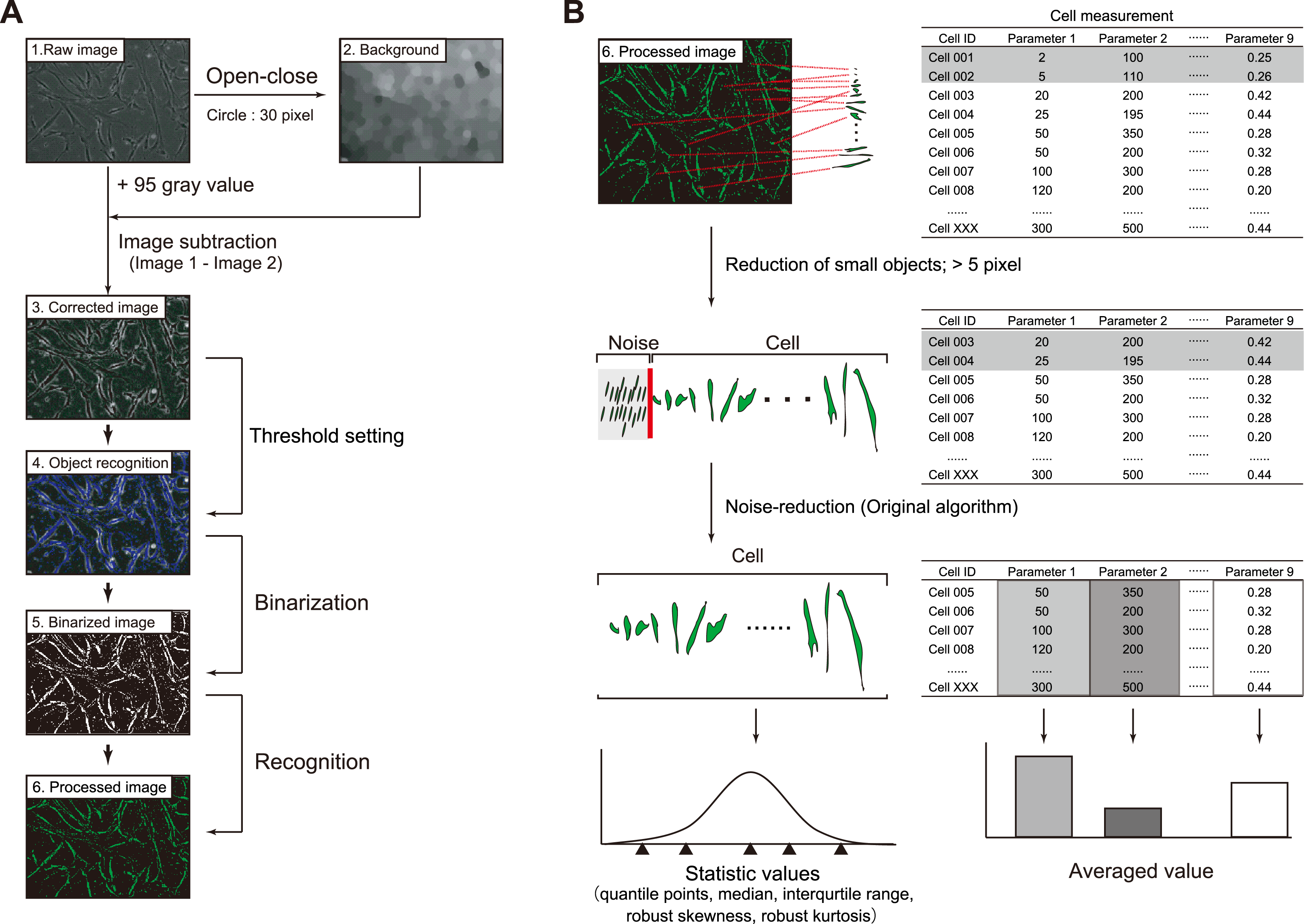

Supplement: Figure S2 — Schematic procedure of image processing and data processing. (A) The procedure listed as Filter 1–4 in Table S3 is illustrated. (B) The procedure listed as Filer 5–6 in Table S3 is illustrated. Especially, the illustration describes the detail of cell measurement and their data processing scheme followed by Filter 6 processing. As shown in the figure, all cells in the images are measured as data consist of “group of cells”, and their distribution is used to calculate statistic values to describe such “group of cells”. Through the process, ells are measured individually by morphological indices; however our final morphological features reflect the information of “group of cells”. In other words, our morphological features contain the information of “heterogeneity” of “group of cells”, which strengthen our prediction model. (TIF) [file pone.0093952.s002.tif]

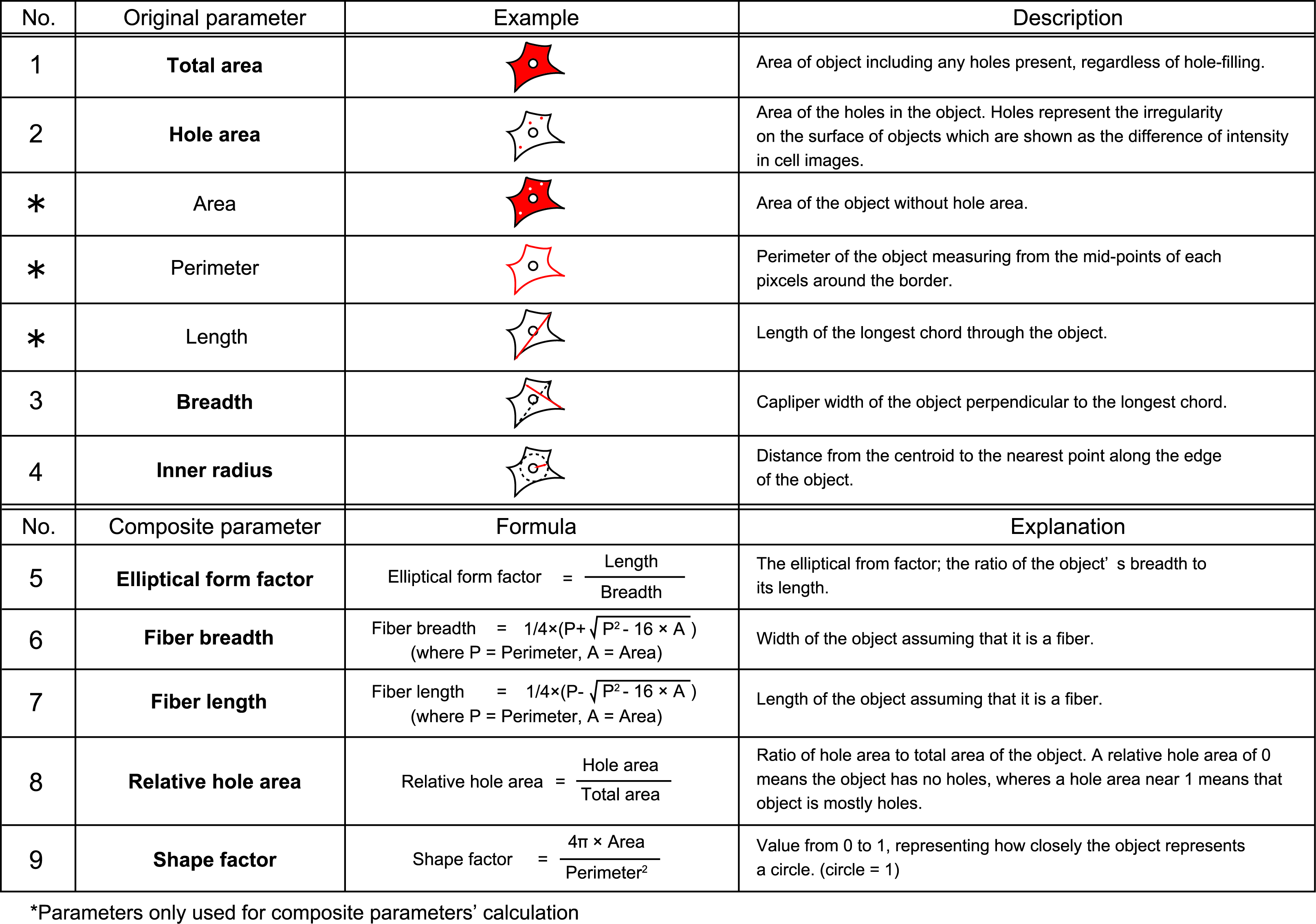

Supplement: Figure S3 — Detailed definition of the basic nine morphological features used for cell measurements. Formulas and schematic illustration of morphological features are presented in detail. The nine features are carefully selected to represent independent information, with the aspect of low-correlating parameters, for stabilizing prediction models which utilize morphological features from label-free phase contrast images. (TIF) [file pone.0093952.s003.tif]

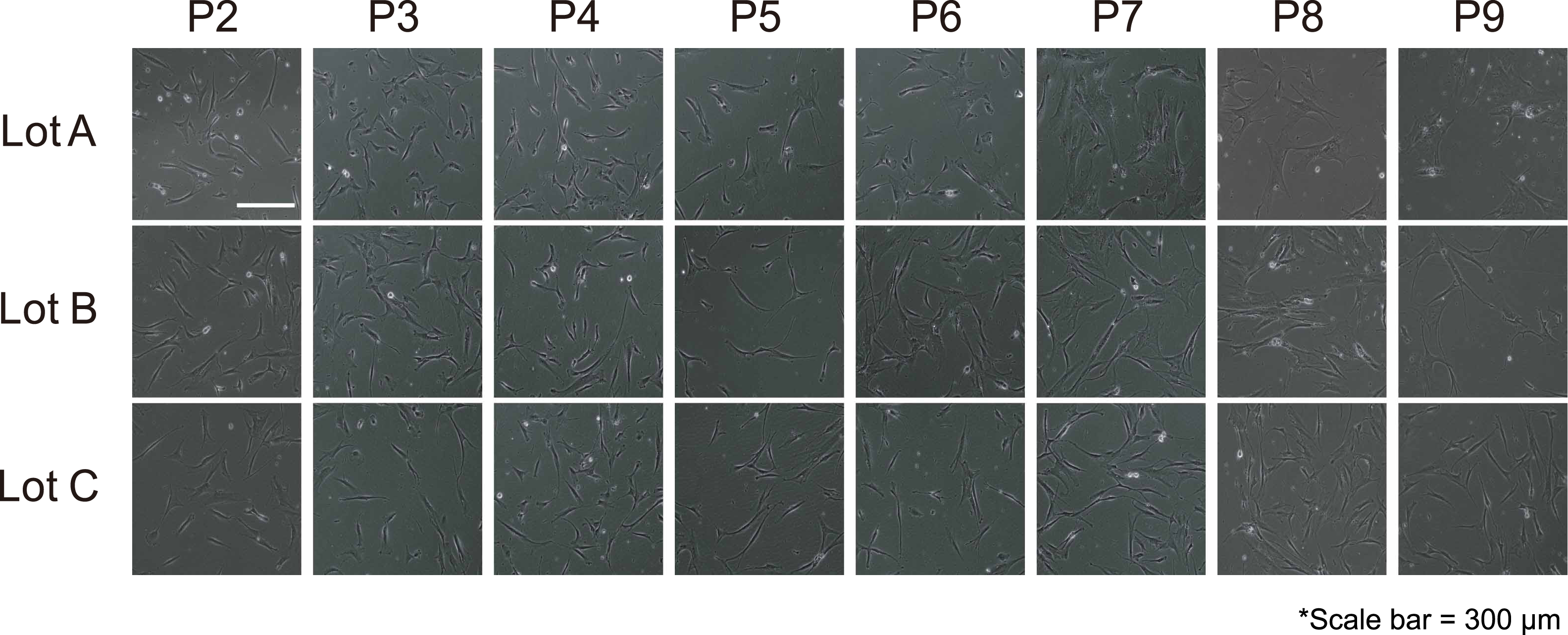

Supplement: Figure S4 — Low-magnification images of cells in Figure 2A . The low-magnification images provides overall image of cellular morphological profile and its distribution. (TIF) [file pone.0093952.s004.tif]
